# Molecular Identification and Expressive Characterization of an Olfactory Co-Receptor Gene in the Asian Honeybee, *Apis cerana cerana*

**DOI:** 10.1673/031.013.8001

**Published:** 2013-08-10

**Authors:** Huiting Zhao, Pengfei Gao, Chunxiang Zhang, Weihua Ma, Yusuo Jiang

**Affiliations:** 1 College of Animal Science and Technology, Shanxi Agricultural University, Taigu Shanxi, 030801, China; 2 Institute of Horticulture, Shanxi Academy of Agricultural Sciences, Taiyuan Shanxi, 030031, China

**Keywords:** gene expression, location analysis, molecular cloning, olfactory receptor

## Abstract

Olfaction recognition process is extraordinarily complex in insects, and the olfactory receptors play an important function in the process. In this paper, a highly conserved olfactory co-receptor gene, *AcerOr2* (ortholog to the *Drosophila melanogaster Or83b),* cloned from the antennae of the Asian honeybee, *Apis cerana cerana* Fabricius (Hymenoptera: Apidae), using reverse transcriptase PCR and rapid amplification of cDNA ends. The full-length sequence of the gene was 1763 bp long, and the cDNA open reading frame encoded 478 amino acid residues, including 7 putative transmembrane domains. Alignment analysis revealed that *AcerOr2* shares high homology (> 74%) with similar olfactory receptors found in other Hymenoptera species. The amino acid identity with the closely related species *Apis mellifera* reached 99.8%. The developmental expression analysis using quantitative real-time reverse transcriptase PCR suggested that the *AcerOr2* transcript was expressed at a relatively low level in the larval stage, whereas it was expressed broadly in the pupal and adult stages, with a significantly high level on the days just before and after eclosion. *In situ* hybridization showed that *AcerOr2* mRNA was expressed in sensilla placodea and on the basal region of the worker antennal cuticle, in accordance with the previous conclusions that the conserved genes are expressed in most olfactory receptor neurons.

## Introduction

Insect olfaction plays a vital role in various life processes, such as mating, ovipositing, avoiding predators, searching for food, and communicating with other members of the species. In insect antennae, the olfactory receptors (ORs) are expressed in many dendritic membranes of the olfactory sensory neurons, participating in the olfaction detection. Seven transmembrane (TM) domain receptor proteins transform the chemical signal to stimulate the neurons. Insect ORs adopt a reverse membrane of G protein-coupled receptors different from the topology structure in mammals (Benton et al. 2006; Ludin et al. 2007).

Olfactory receptors in insects are highly diverse except for the Or83b family. The family shares a highly conserved gene sequence with different insects and plays an important role in regulating insect behavior (Gao and Chess 1999; Larsson et al. 2004; Jones et al. 2005; Wicher et al. 2008). In *Drosophila,* Or83b forms a heterodimer with other ORs and tends to act as a co-receptor (Neuhaus et al. 2005; Benton et al. 2006). *Or83b* mutant flies did not show odorant-evoked action potential with slight spontaneous activity (Larsson et al. 2004; Tsitoura et al. 2010). Hence, the Or83b family plays a crucial role in detecting odorants or pheromones. Recently, *Or83b* was renamed *Oreo,* the acronym for olfactory receptor co-receptor (Vosshall and Hansson 2011). The conservative genes from species of Diptera, Lepidoptera, Hymenoptera, and Coleoptera have been identified based on their homology (Hill et al. 2002; Krieger et al. 2003; Melo et al. 2004; Xia and Zwiebel 2006; Malpel et al. 2008; Jordan et al. 2009; Shen et al. 2011).

*AmelR2,* the *DOr83b* orthologue gene in *Apis mellifera,* has been cloned and located (Krieger et al. 2003); however, the expression pattern of *AmelR2* has not yet been fully described in detail. The Asian honeybee, *A*pis *cerana cerana* Fabricius (Hymenoptera: Apidae), is an indigenous honeybee species in China. In our study, we report the molecular and expressive characterization of *AcerOr2,* a new *Oreo* gene in *A. cerana,* to provide a basis for further research on the function of *AcerOr2 m A. c. cerana.*

## Materials and Methods

### Samples

Bees were reared at the apiary of Shanxi Agriculture University, Shanxi, China. The thorax and the antennae of adult worker bees were collected for separate PCR analyses of the corresponding DNA and RNA sequences. Tissues obtained from different stages, larval (6 whole bodies), pupal (6 heads), and adult (30 pairs of antennae), were dissected and used directly in isolating the total RNA for the expression profiling of worker bees at different ages.

### DNA and RNA extraction

Total DNA was extracted from the thorax of each individual using a modified method described by Smith and Hagen (1996). Total RNA was isolated from 50 antennae of adult worker bees using Trizol reagent (Invitrogen, www.invitrogen.com), and stored at -70° C until they were used.

### Genome internal amplification

cDNA was synthesized from the total RNA isolated from the antennae using a PrimerScript RT reagent Kit (TaKaRa, www.takarabio.com). Specific primers were designed based on the sequences closely related to the gene *AmelOr2* (DQ449670) for *AcerOr2* DNA and cDNA amplification. DNA primers were: Forwardl: 5′ TCACCATGCTCTTCTTCACG- 3′-, Reversel: 5′ - CGCTGAATTCCATCAAAGGC- 3′; and F2: 5′ TGCTCGTGGCTCCTGTTCGC- 3′, R2: 5′ - AGCAGTTGGCCGGAAGGTGG- 3′. Samples were subjected to pre-denaturation for 4 min at 94° C first, then 30 cycles of amplification were performed at 94° C for 30 sec, 55° C for 32 sec, and 72° C for 1 min, followed by incubation at 72° C for 8 min. The cDNA primers were: F: 5′ AAGACGTGGACGATCTCACC- 3′ and R: 5′ -GCTACACCATAGGCGTCTCC- 3′). PCR reactions were performed as follows: after 3 min at 94° C, 35 cycles for 30 sec at 94° C, 1 min at 56° C and 1 min at 72° C, then an 8 min elongation at 72° C. PCR products were analyzed by 1.5% agarose gel electrophoresis.

### Rapid amplification of cDNA ends (RACE) amplification

cDNAs were synthesized using the SMART RACE cDNA amplification kit (Clontech, www.clontech.com) for the 3′ and 5′ RACE. The 3′ and 5′ regions of the cDNAs were obtained using 3′ and 5′ RACE-PCRs following the manufacturer's instructions. Gene-specific primers (GSPs) were designed based on the sequence of internal PCR products. 5′ 'RACE GSP: 5′ CCCTCCAGCTCCTCGCAGAGTCATGCC G- 3′; 3′ RACE GSP: 5′ GCCAATGGTATGATGGCTCCGAGGAAG CC- 3′. Amplification reactions were carried out as follows: 94° C for 3 min; then 33 cycles at 94° C for 30 sec, 58∼60° C for 40 sec, and 72° C for 1 min, and 72° C for 6 min.

### Cloning and sequencing

The PCR products were cloned and sequenced in Huada Gene Research Center (Beijing, China). The overlapping sequences obtained from two internal amplification fragments of DNA were combined to produce the *AcerOr2* DNA; similarly, the internal amplification segment of cDNA and the 3′, 5′ RACE products were combined to produce the *AcerOr2* mRNA Homology searches were conducted on the NCBI platform (www.ncbi.nlm.nih.gov). Alignments of multiple sequences were carried out using ClustalW (Thompson et al. 1994). The gene phylogenetic tree was built on Mega 4.0 software (www.megasoftware.net) according to neighbor-joining algorithms (Tamura et al. 2007).

### Developmental expression by real-time quantitative PCR (qRT-PCR)

The total RNA of worker bees, larvae (2, 4, and 6 days old), pupae (5 and 10 days old), and adults (1, 5, 10, 15, 20, 25, 30, 35, and 40 days old) for expression profiling were extracted. The corresponding partial cDNA sequences were amplified with the primers designed on the complete sequence of *AcerOr2* (F: 5′ GGATCAGAGGAGGCCAAAAC- 3′, R: 5′ - CCAACACCGAAGCAAAGAGA- 3′) *RPS18* was used as a housekeeping gene based on A *mellifera RPS18* (Scharlaken et al. 2008) (F: 5‘ GATTCCCGATTGGTTTTTGA- 3′; R: 5′ - CCCAATAATGACGCAAACCT- 3′). qRT-PCR was run on M×3000P real-time PCR system (Stratagene, www.stratagene.com) using the SYBR Premix Ex Taq kit (Takara). All samples were tested in triplicate. The reactions were incubated in a 96-well plate at 95° C for 20 sec, followed by 45 cycles at 95° C for 15 sec and at 60° C for 20 sec. A dissociation curve was created with the thermal profile at 95° C for 30 sec, 60° C for 30 sec, and 95° C for 30 sec. The 2
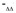
^Ct^ method was used to measure the relative expression levels of the samples (Livak andSchittgen 2001).

### mRNA in situ hybridization

Three digoxigenin-labelled RNA antisense oligonucleotide probes were synthesized by Boster (www.bosterbio.com) according to the cDNA full-length of *AcerOr2,* which was acquired in the study. The sequence of the antisense probes was as follows:


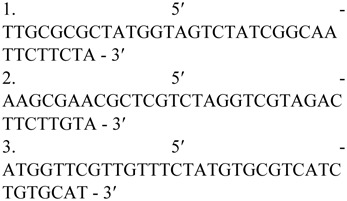


Antennae from 1- to 3-day-old adult workers were dissected, embedded in Tissue-Tec OCT Compound (Sakura, www.sakura.eu). Cryosections (8 µm) were prepared at -22° C and mounted on poly-L-lysine treated slides. Samples were air-dried for 5 min before being fixed in 4% paraformaldehyde/O. 1M phosphate-buffered saline (PBS) with 0.1% diethyl pyrocarbonate. Then, the slides were washed 3 times with double distilled water and airdry ed for about 30 min. The hybridization protocol was performed according to the RISH kit instructions (Boster). In the process, slides were prehybridized for 3 hr. Then, hybridization solution was added to the tissue section, and each slide was covered with a coverslip. Slides were hybridized for 16 hr with the digoxigenin-labeled antisense probes at 42° C in a humid box with 20% glycerol (double distilled water was substituted for the antisense probes as a negative control). After hybridization, the coverslip was removed, and slides were washed twice in 2×SSC for 5 min at 37° C, and once in 0.5×SSC and 0.2×SSC for 15 min at 37° C respectively. Then, they were treated with blocking solution for 30 min at 37° C and washed 4 times in 1×PBS for 5 min. Slides were incubated in biotinylation mouse anti-digoxigenin for 1 hr at 37° C and then in Strept Avidin-Biotin Complex- alkaline phosphatase for 30 min at room temperature. After being washed 4 times in 1×PBS for 5 min, the hybridization signals were visualized using nitroblue tetrazolium and 5-brom-4-chlor-3-indolyl. Finally, the sections were viewed by using a light microscope (Olympus BX53, www.olympusglobal.com) and were imaged using ImagePro Plus 7.0 software (Media Cybernetics, www.mediacy.com).

## Results

### Characterization and analysis of the partial DNA fragments

*AcerOr2,* a fragment of 1138 bp, was obtained by splicing the 2 internal specific overlapping PCR sequences. The fragment contained 3 exons and 2 introns, with 58.9% A+T nucleotides and 41.1% G+C nucleotides.

**Figure 1.**
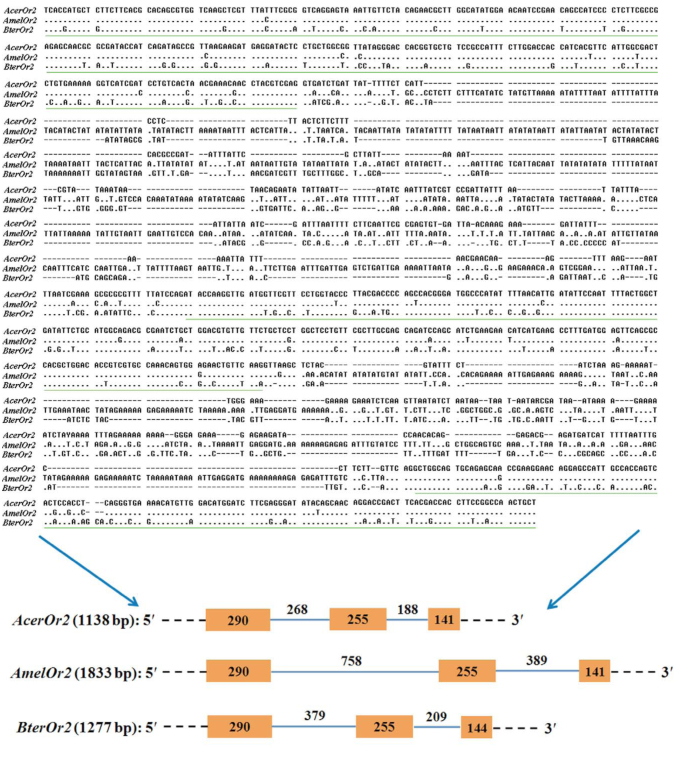
Alignment of the partial DNA sequence of *AcOr2* with its homologous sequence of other insects. represent the same base , represent the missing base, sequences above the underline are exons. The arrows point to the ideograph of the above alignment sequences. Figures inside the boxes and above the lines correspond to the lengths of the exons and introns, respectively. *Acer, Apis cerana* (JN544931 ); *Amel, Apis mellifera* (NC_007070); *Bter, Bombus terrestris* (NW_003566036). High quality figures are available online.

Alignment with the other 2 homologous DNA sequences (BLAST in NCBI) revealed that *AcerOr2* exhibits higher identity with *Amel0r2* (80.4%) than with *BterOr2* (72.6%). Respectively, *AmelOr2* and *BterOr2* exhibit 96%/64.8% and 83%/62.2% similarity with the exons/introns of *AcerOr2.* The divergence was mainly attributed to the difference in length of the introns and the nucleotide variation. The alignment sequences and the ideograph of the 3 genes are shown in Figure 1.

### Characterization and analysis of the fulllength cDNA

**Figure 2.**
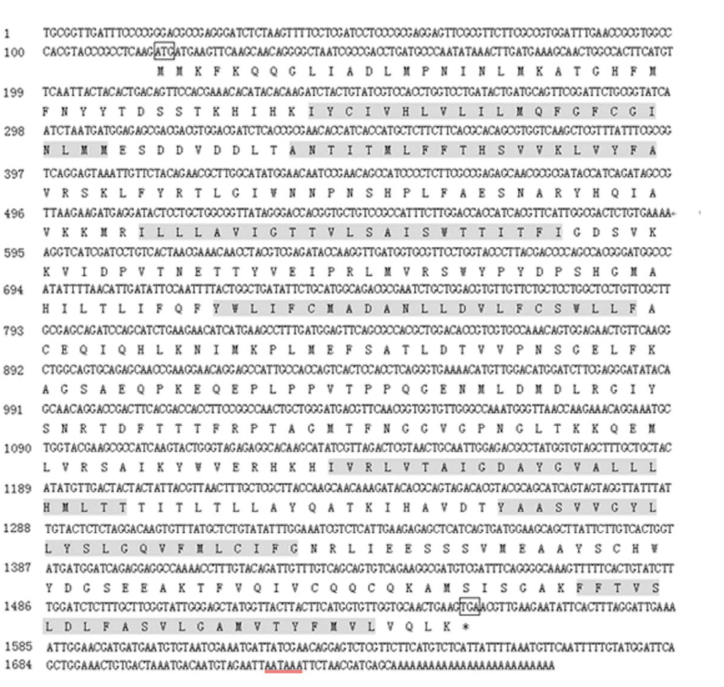
The nucleotide and deduced amino acid sequences of *AcerOr2.* The boxed codons represent the start and stop codons. Double-red underline represents the polyadenylation signal. The shaded sequences represent the TM domains. High quality figures are available online.

Using RT-PCR and RACE methods, identified the full-length cDNA sequence of *AcerOR2* was identified (GenBank accession number: JN792581) (Figure 2). Sequence analysis showed that the open reading frame was 1437 bp long and encodes a protein comprising 478 amino acids with a molecular weight of 53.81 kDa. The 5′ and 3′ noncoding regions contained 116 bp and 210 bp, respectively. *AcerOR2* was predicted to contain 7 TM topology structures of ORs (determined by TMHMM 2.0, Krogh et al. 2001).

**Figure 3.**
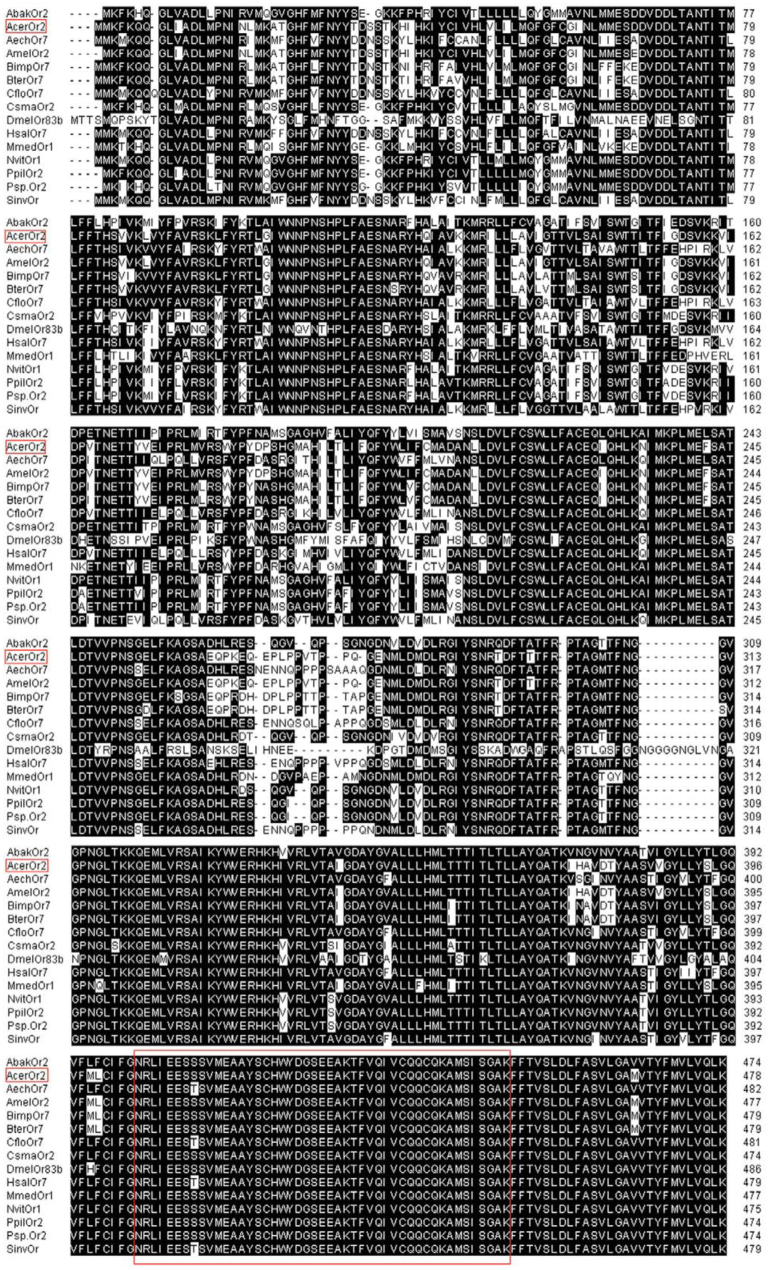
Alignment of the deduced amino acid sequence of *AcerOr2* with other selected members of *Orco* family. Identical residues are shaded in black. Dashed lines indicate the gaps. The amino acids in the red box represent the most conserved domain between TMs Vl and VII. Abak, *Apocrypta bakeri* (ABY51615); Acer, *Apis cerana* (AET85154); Aech, *Acromyrmex echinatior* (EG163650); Amel, *Apis mellifera* (NP_001128415); Bimp, *Bombus impatiens* (XP_003494153); Bter, *Bombus terrestris* (XP_003402775); Cflo, *Camponotus floridanus* (EFN70194); Csma, *Ceratosolen solmsi marchait* (ABY51614); Dmel, *Drosophila melanogaster* (AAT71306); Hsal, *Harpegnathos saltator* (EFN84180); Mmed, *Microplitis mediator* (ABM05966), Nvit: *Nasonia vitripennis* (NP_001164465); Ppil, *Philotrypesis pilosa* (ABY51616); Psp., *Philotrypesis* sp.(ABY5l6l7); Sinv, *Solenopsis invicta* (EFZ15266). High quality figures are available online.

**Figure 4.**
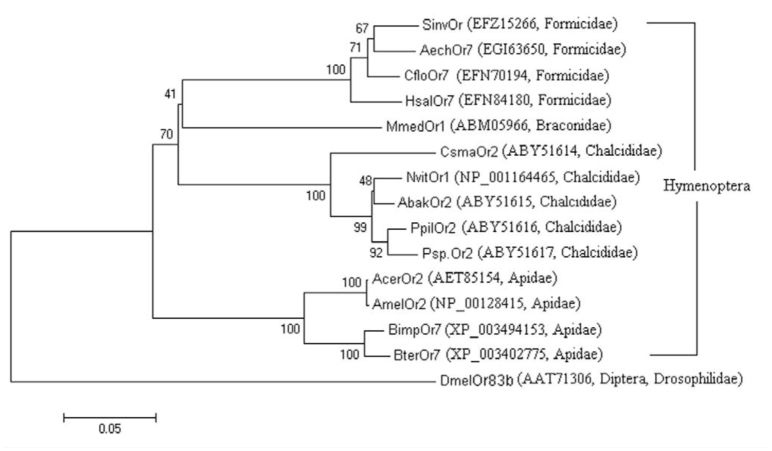
Phylogenetic relationships of the *Orco* family in Hymenoptera. The branch labels are bootstrap values. Bootstrap support values (%) are based on 1,000 replicates. Scale bar is 0.05. High quality figures are available online.

The alignment of amino acid sequence of AcerOr2 with DmelOr83b and other orthologues of Hymenoptera (Figure 3) indicated that AcerOr2 shared high levels of conservation with other Hymenoptera *Orco* genes (> 74% identity). It showed a considerable similarity to AmelOr2 (99.8% identity) and a relatively low similarity to DmelOr83b (64.9%). Isogeny in Hymenoptera was similar with that in Lepidoptera (Xiu et al. 2010). The alignment also revealed a highly conserved region in the C-terminus, particularly between TMs VI and VII, which is remarkably identical in all of the species. To investigate and confirm the evolutionary relationships among these alignment species, a phylogenetic tree was constructed using the neighbor-joining method (Figure 4). The tree showed that the selected Hymenoptera insects were grouped into 4 main clusters or families, namely Apidae, Formicidae, Braconidae, and Chalcididae.

### Developmental expression pattern in worker bees

**Figure 5.**
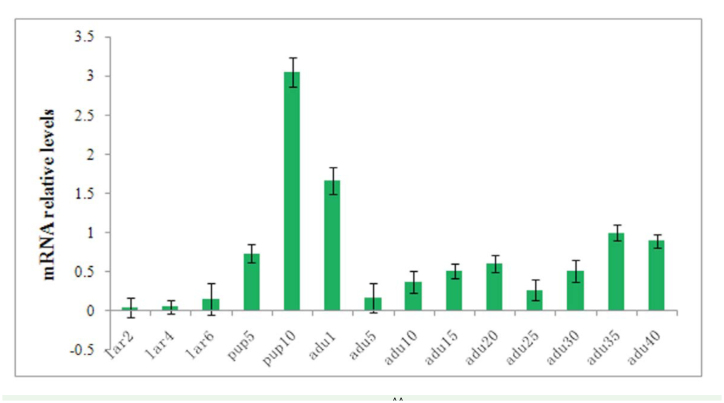
*AcerOr2* gene expression in *Apis cerana cerana* antennae. The 2
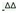
^CT^ method was used to measure the relative expression levels of the samples. The expression ratios were first normalized to the control gene *RPS18,* and then normalized on the 30th day in each trial. Lar2 and lar4 represent the 2- and 4-day-old uncapped larvae, respectively. Lar6 represents the 6-day-old capped larvae. Pup5 and pup 10 represent the 5- and 10-day-old pupae, respectively, adul to adu40 represent the days after eclosion. Data are shown as mean ±SEM. High quality figures are available online.

The expression pattern of *AcerOr2* transcripts was determined by qRT-PCR on different developmental stages in worker bees (Figure 5). qRT-PCR results showed that *AcerOr2* transcripts were expressed broadly in adults. The expressions were expressed only slightly in larvae, and as the developmental stages progressed, the expression level increased. Notably, the expression reached the maximum level on the day prior to eclosion, revealing that the ability for olfactory recognition enhanced sharply. Furthermore, there was a relatively high level of expression on the first day after eclosion.

### Location analysis in antennae of worker bees

**Figure 6.**
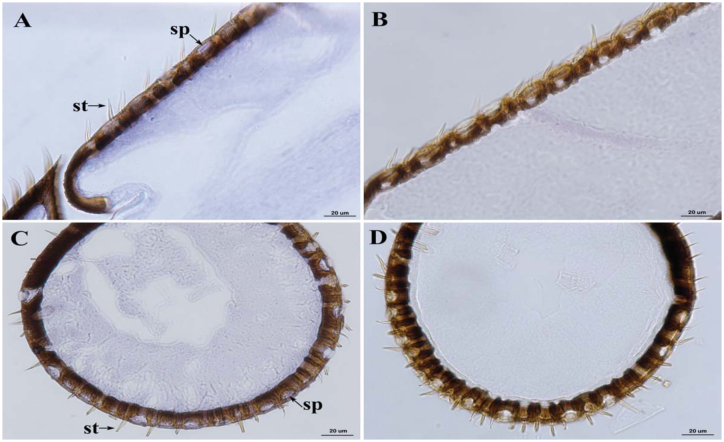
The distribution of *AcerO2* transcripts in worker antennae of *Apis cerana cerana.* A, B are longitudinal and cross section with DIG-labeled probes to *AcerOr2* (×40). Positive signals are in dark blue. C, D are controls with no labeling (×40). sp, sensilla placodea; st, sensilla trichoidea. Scale bar: 20 µm. High quality figures are available online.

There are several types of chemosensilla in the antennae of honeybee (Esslen and Kaissling 1976), and the sensilla placodea and sensilla trichoidea may be associated with olfactory sensation (Sliber and Sekhon 1961; Kaissling and Renner 1968). In our study, the expression pattern of *AcerOr2* was investigated in workers' antennae. The positive signals were distributed in most sensilla placodea and on the basal region of the antennal cuticle (Figure 6). The expression location of the mRNA transcripts was similar to that found in *AmelOr2* (Krieger et al.2003). No signal was detected in the negative control section.

## Discussion

The ORs of insects are highly diverse and share almost no identity with other vertebrates and nematodes (Gao and Chess 1999; Smith 1999). In OR superfamily, the *Orco* family is unique. The family contains the highly conserved OR proteins with 60% to 80% amino acid identity shared among the divergent insect species (Larsson et al. 2004; Patch et al. 2009).

In the current study, *AcerOr2,* a putative OR gene, was identified from the *A. c. cerana.* The AcerOr2 protein exhibited similar characteristics with other Or83b orthologues. For instance, it comprised a predicted 7-TM domain structure and shared a high sequence identity with other *Orco* genes. Moreover, *AcerOr2* contained the remarkably conserved motif domain spans TMs VI and VII, which has been demonstrated to be at least part of the interaction domain between the conventional ORs and the co-receptor in *D. melanogaster* (Benton et al. 2006). Thus, we deduce that *AcerOr2* belongs to the *Orco* subfamily.

DOr83b and the corresponding orthologs represent the highly conserved protein family, as observed in our study. However, different species exhibit a distinct variation in the introns, which may be primarily attributed to the insertion or the deletion of a base. Although the insects belong to the same order, comparing the similarity among the insects is difficult because of the variation in their introns. The result indicates that the *Or2* gene in *A. cerana,* perhaps including other members of *Orco* family, is evolving quickly, as showed in the study of the *Or2* gene in *Ceratosolen solmsi* (Lu et al. 2009).

The qRT-PCR results revealed that *AcerOr2* transcripts were expressed in all developmental stages, especially in pupae and adults. The faint expression level was tested in the larvae, as observed in *D. melanogaster* (Kreher et al. 2005), *Culex quinquefasciatus* (Xia and Zwiebel 2006), *Spodoptera littoralis* (Malpel et al. 2008), and *Bactrocera cucurbiae* (Shen et al. 2011), indicating that the ability of olfactory identification may be very weak. Two expression peaks appeared on the day just before and after eclosion. This result might be attributed to the response mechanism to chemical stimuli for the environmental change during eclosion. In the *in situ* experiment, labeling of DIG-labeled antisense probes to *AcerOr2* was observed in the sensilla placodea and numerous cells located near the basement membrane of the antennae. The numerous cells were likely housed in the sensilla placodea and sensilla trichoidea. The expression pattern of *AcerOr2* was similar to other studies about the Orco members, such as *DOr83b, AmelR2, AgOr7,* and *MsexOr2* (Vosshall et al. 1999; Krieger et al. 2003; Pitts et al. 2004; Malpel et al. 2008; Patch et al. 2009). The broad expression profile of *Orco* family members suggests that these olfactory receptors may play an extensive role in olfactory recognition, such as receiving kinds of pheromones and foraging for nectar, pollen, or other odorants. The labeled sensilla, sensilla placodea and sensilla trichoidea, have been described to be associated with olfactory sensation (Sliber and Sekhon 1961; Kaissling and Renner 1968). So, *AcerOr2* might play a role in odorant perception of *A. cerana cerana.*

Several studies have verified that *Or83b* is necessary olfactory responses in *Drosophila* (Larsson et al. 2004; Benton et al. 2006). It can also be co-expressed with a conventional OR to form a ligand-gated cation channel (Sato et al. 2008; Wicher et al. 2008; Jones et al. 2011). The new signal transduction model of insects induced more interest in the study of the relationship between the co-expressed ORs. As a native Chinese bee species, *A. cerana cerana* appears to exhibit highly sensitive olfactory receptors. Based on the identification of the new Hymenoptera *Orco* genes described in this study, further research on ORs have been developed to elucidate the interacting mechanism of the co-receptors and the superior ability of *A. cerana cerana* in searching for nectar and pollen.

## Abbreviations

**GSP**,: gene specific primer
OR,: olfactory receptor
**RACE**,: rapid amplification of cDNA ends
**qRT-PCR**,: real time quantitative reverse transcriptase PCR
**RT-PCR**,: reverse transcriptase PCR
**TM**,: transmembrane
